# Fibrin membrane pupillary-block glaucoma after uneventful cataract surgery treated with intracameral tissue plasminogen activator: a case report

**DOI:** 10.1186/1471-2415-12-3

**Published:** 2012-03-20

**Authors:** Hideaki Yoshino, Masaaki Seki, Jun Ueda, Takaiko Yoshino, Takeo Fukuchi, Haruki Abe

**Affiliations:** 1 Division of Ophthalmology and Visual Science, Graduate School of Medical and Dental Sciences, Niigata University, 1-757 Asahi-machi, Niigata-shi 951-8120, Japan

**Keywords:** Pupillary block glaucoma, Fibrin membrane, Cataract surgery, Anterior segment imaging, Tissue plasminogen activator

## Abstract

**Background:**

Fibrin pupillary-block glaucoma is a rare complication after cataract surgery. The treatment for this condition is still controversial, since Nd:YAG laser fibrin membranotomy tends to reocclude and laser peripheral iridotomy entails the risk of damaging the corneal endothelium in the presence of corneal edema associated with elevated intraocular pressure.

**Case presentation:**

A 62-year-old man with diabetes mellitus developed acute elevation of intraocular pressure with a shallow anterior chamber five days after uneventful cataract surgery. Initially, slit lamp examination provided only limited information due to severe corneal edema. After resolution of corneal edema with systemic glaucoma therapy, a complete fibrin membrane was observed across the pupil by slit lamp examination. Anterior segment optic coherence tomography clearly revealed a thin fibrin membrane covering the entire pupillary space, a shallow anterior chamber, and a deep posterior chamber. The intraocular lens was not observed by anterior segment optic coherence tomography. In contrast, ultrasound biomicroscopy, which has superior penetration depth, was able to visualize the intraocular lens deep in the posterior chamber. Injection of tissue plasminogen activator into the anterior chamber resulted in complete fibrinolysis and released the pupillary block.

**Conclusion:**

This case suggests that ocular anterior segment imaging modalities, especially ultrasound biomicroscopy, serve as powerful diagnostic tools to identify mechanisms of acute angle closure glaucoma, which is often accompanied by poor intraocular visibility. This is the first reported case of fibrin pupillary-block glaucoma after cataract surgery successfully treated with intracameral tissue plasminogen activator.

## Background

Fibrin membrane pupillary-block glaucoma is an uncommon complication after phacoemulsification cataract surgery [1]. Severe corneal edema due to elevated intraocular pressure often prevents a thorough slit lamp examination, making early diagnosis difficult. Corneal edema similarly interferes with laser iridotomy. We present here a case of fibrin membrane pupillary-block glaucoma that was diagnosed with help of ocular anterior segment imaging devices and successfully treated with tissue plasminogen activator.

## Case presentation

The patient was a 62-year-old Japanese man with diabetes mellitus on insulin therapy for 20 years. He visited an ophthalmology unit in a general hospital to check for diabetic retinopathy. His visual acuity was 0.5 in the right eye and 0.3 in the left eye. Intraocular pressure (IOP) was 13 mmHg and 14 mmHg in the right and left eyes, respectively. The anterior chambers were deep and clear in both eyes. Examination revealed cortical cataract and pre-proliferative diabetic retinopathy in the both eyes. Uneventful phacoemulsification cataract surgery with in-the-bag intraocular lens (IOL) implantation was performed in the left eye on March 10, 2011, when his blood glucose level was 98 mg/dl, and his HbA_1_c was 7.4%. On the next day, the visual acuity was 0.8, and IOP was 15 mmHg. The anterior chamber was deep with only mild inflammation (1+ cellular reaction). On postoperative day 5, the patient suffered sudden ocular pain in the operated eye and repeatedly vomited. The visual acuity in the left eye was 0.02, and IOP was 68 mmHg. The anterior chamber was shallow, but severe corneal edema prevented further detailed slit lamp examination. The patient was treated with instillation of topical glaucoma medication (0.5% timolol maleate) and systemic mannitol. IOP remained high in the 60 mmHg range despite intensive anti-glaucoma therapy. A physician suspected the presence of a thin membrane covering the pupil and thus attempted Nd:YAG laser membranotomy, which was unsuccessful due to corneal edema.

**Figure 1:**
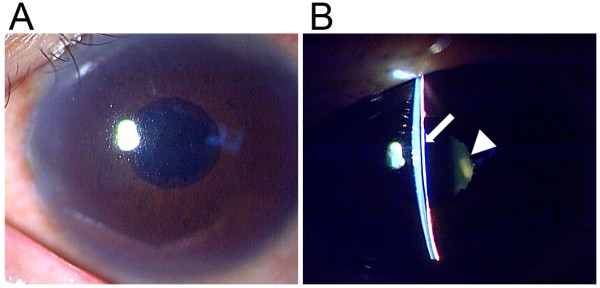
**Slit lamp examination on the day of transfer to our hospital (postoperative day 6)**. (**A**) Corneal edema resolved after systemic glaucoma therapy. (**B**) The anterior chamber was shallow, resulting in peripheral iridocorneal contact over the entire circumference. A thin fibrin membrane covered the pupil (arrow) while the IOL was located at a distance from the pupil (arrowhead).

**Figure 2:**
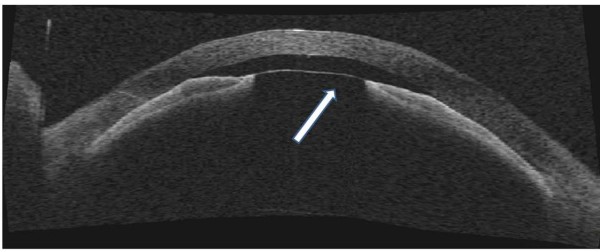
**Anterior segment OCT on the day of admission to our hospital (postoperative day 6)**. The anterior chamber was very shallow (0.46 mm). Anterior segment OCT clearly revealed a fibrin membrane across the pupil (arrow) and iridocorneal contact. The IOL was not observed by anterior segment OCT. Note the deep posterior chamber.

**Figure 3:**
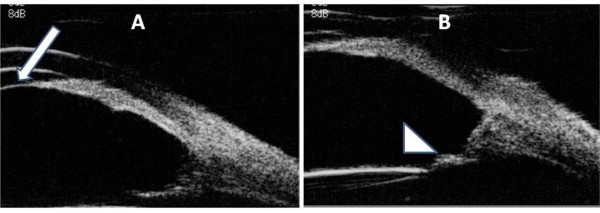
**UBM on the day of admission to our hospital (postoperative day 6)**. **(A) **The fibrin membrane across the pupil was revealed by UBM (arrow). The posterior chamber was extremely deep. **(B) **UBM localized the posterior chamber IOL (arrowhead) at a distance from the pupil. The ciliary sulcus was very wide, ruling out the possibility of malignant glaucoma.

**Figure 4:**
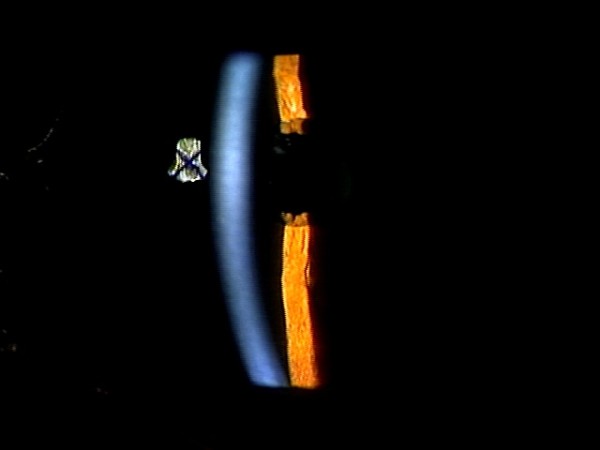
**Slit lamp examination one hour after intracameral tPA injection**. The fibrin membrane had lysed, and the anterior chamber deepened.

When the patient was transferred to Department of Ophthalmology in Niigata University Hospital on postoperative day 6, IOP was reduced to 16 mmHg in the left eye, and the cornea became clear (Figure 1A). Slit lamp examination revealed a thin membrane covering the pupill, and the IOL was positioned at a distance from the pupil (Figure 1B). Anterior segment optical coherence tomography (AS-OCT) (SL-OCT; Heidelberg Engineering, Heidelberg, Germany) (Figure 2) and ultrasound biomicroscopy (UBM) (Humphrey UBM model 840; Carl Zeiss Meditec, Dublin, CA) (Figure 3) further confirmed a complete fibrin membrane across the pupillary space. While the anterior chamber was shallow, the posterior chamber was deep with a wide ciliary sulcus, ruling out the possibility of malignant glaucoma (Figures 2 and 3). Although the IOL was not visualized with AS-OCT, UBM, which has superior penetration, clearly located the IOL deep in the posterior chamber. We made a diagnosis of pupillary-block glaucoma due to a complete fibrin membrane, and injected 25 μg of tissue plasminogen activator (tPA) intracamerally. Usage of tPA was a part of standard care for postoperative fibrin reaction at our hospital. Within an hour, the fibrin membrane was lysed (Figure 4). The anterior chamber became deep, with IOP of 16 mmHg. On the day following tPA injection, IOP became elevated to 50 mmHg presumably due to a response to topical steroids, since IOP returned to the 10 mmHg range after tapering steroid therapy. His ocular findings in the left eye as of April 26, 2011 were as follows: visual acuity, 0.9; IOP, 12 mmHg.

## Discussion

The differential diagnosis for a shallow anterior chamber associated with elevated IOP after cataract surgery includes pupillary block (fibrin membrane [1], Soemmering's ring [2], and posterior synechia [3]), capsular block syndrome [4], and malignant glaucoma [5]. Although fibrin membrane pupillary-block glaucoma is more common after pars plana vitrectomy [6,7], there are a few reports of fibrin membrane pupillary-block glaucoma after cataract surgery [1,8].

Detection of a thin fibrin membrane by conventional slit lamp examination is very difficult in the presence of severe corneal edema associated with elevated IOP. In contrast, UBM can visualize the anterior segment even under poor intraocular visibility due to its echographic nature. If UBM had been available in the acute phase of IOP elevation in the present case, an immediate diagnosis would have been possible, leading to earlier treatment. However, in the present case, the fibrin membrane was observed only after the cornea became clear following intensive glaucoma treatment including systemic mannitol, which resulted in acute renal dysfunction in addition to the patient's diabetic nephropathy. As demonstrated in this case report, AS-OCT is a useful diagnostic tool, but can only provide limited information when the cornea is hazy. Moreover, since AS-OCT has less penetration depth than UBM, it is difficult to examine the ciliary body, which is essential in the evaluation of the possibility of malignant glaucoma. In the present case, AS-OCT failed to visualize the IOL located deep in the posterior chamber for the same reason. We thus believe UBM has the strongest diagnostic utility for acute angle closure as previously reported [9].

So far hypertension and diabetes mellitus have been proposed as risk factors of fibrin formation after cataract surgery [8,10]. In a large cohort study [8], accumulation of fibrin after extracapsular cataract extraction was seen more often in patients with diabetic retinopathy, but only one patient with diabetes mellitus developed fibrin pupillary block. Kohr *et al. *reported fibrin pupillary-block glaucoma after phacoemulsification cataract surgery in four patients, three of whom had diabetes mellitus [1]. Previous reports suggested that preposition to breakdown of the blood-aqueous barrier in diabetics is associated with fibrin formation after cataract surgery [10,11]. Diabetes mellitus was also present in the present patient.

The treatment of fibrin pupillary-block glaucoma has not been established. In cases following pars plana vitrectomy, it is often treated by laser peripheral iridotomy [6] or intraocular tPA (25 μg) [7]. In a case series of fibrin pupillary-block glaucoma after phacoemulsification cataract surgery, all patients were successfully treated by Nd:YAG laser peripheral iridotomy [1]. Two cases underwent Nd:YAG fibrin membranotomy, but the fibrin membrane resealed in one patient [1]. Nd:YAG laser iridotomy is a viable treatment option for fibrin pupillary block, but it is technically difficult and has the potential risk of damaging the corneal endothelium when there is severe corneal edema or iridocorneal contact. In a randomized prospective study of patients with fibrin accumulation in the anterior chamber after cataract surgery, a single anterior chamber injection of tPA (10 μg) reduced the incidence and quantity of fibrin without any adverse effects [12]. The advantage of anterior chamber tPA injection is that the procedure can be performed safely even in an eye with poor intraocular visibility as long as careful sonographic examination was performed using UBM.

## Conclusions

Physicians have to include fibrin membrane pupillary block in the differential diagnosis for shallow anterior chamber after cataract surgery. UBM provides important diagnostic information even in cases with severe corneal edema. In this case, intracameral tPA injection was used to successfully treat fibrin membrane pupillary block.

## Consent

Informed consent was obtained from the patient for publication of this case report and any accompanying images. A copy of the written consent is available for review by the series editor of this journal.

## Abbreviations

IOP: Intraocular pressure; IOL: Intraocular lens; AS-OCT: Anterior segment optical coherence tomography; UBM: Ultrasound biomicroscopy; tPA: Tissue plasminogen activator.

## Competing interests

The authors declare that they have no competing interests.

## Authors' contributions

HY participated in management of the case, analyzed the data, and drafted the manuscript. MS was the attending surgeon for the case and revised the manuscript. TY participated in management of the case. JU and TF supervised physicians involved in the patient's care and interpreted the data. HA offered administrative and material support. All authors read and approved of the final manuscript.

## Pre-publication history

The pre-publication history for this paper can be accessed here:

http://www.biomedcentral.com/1471-2415/12/3/prepub
